# Supplementary material for: Neuronal Connectivity as a Determinant of Cell Types and Subtypes
Source: Res Sq. 2023 Jun 14:rs.3.rs-2960606. Preprint. [Version 1] doi: 10.21203/rs.3.rs-2960606/v1 (PMC10312949; doi:10.21203/rs.3.rs-2960606/v1)
Supplement: Supplement 1 [file NIHPPRS2960606V1-supplement-1.pdf]

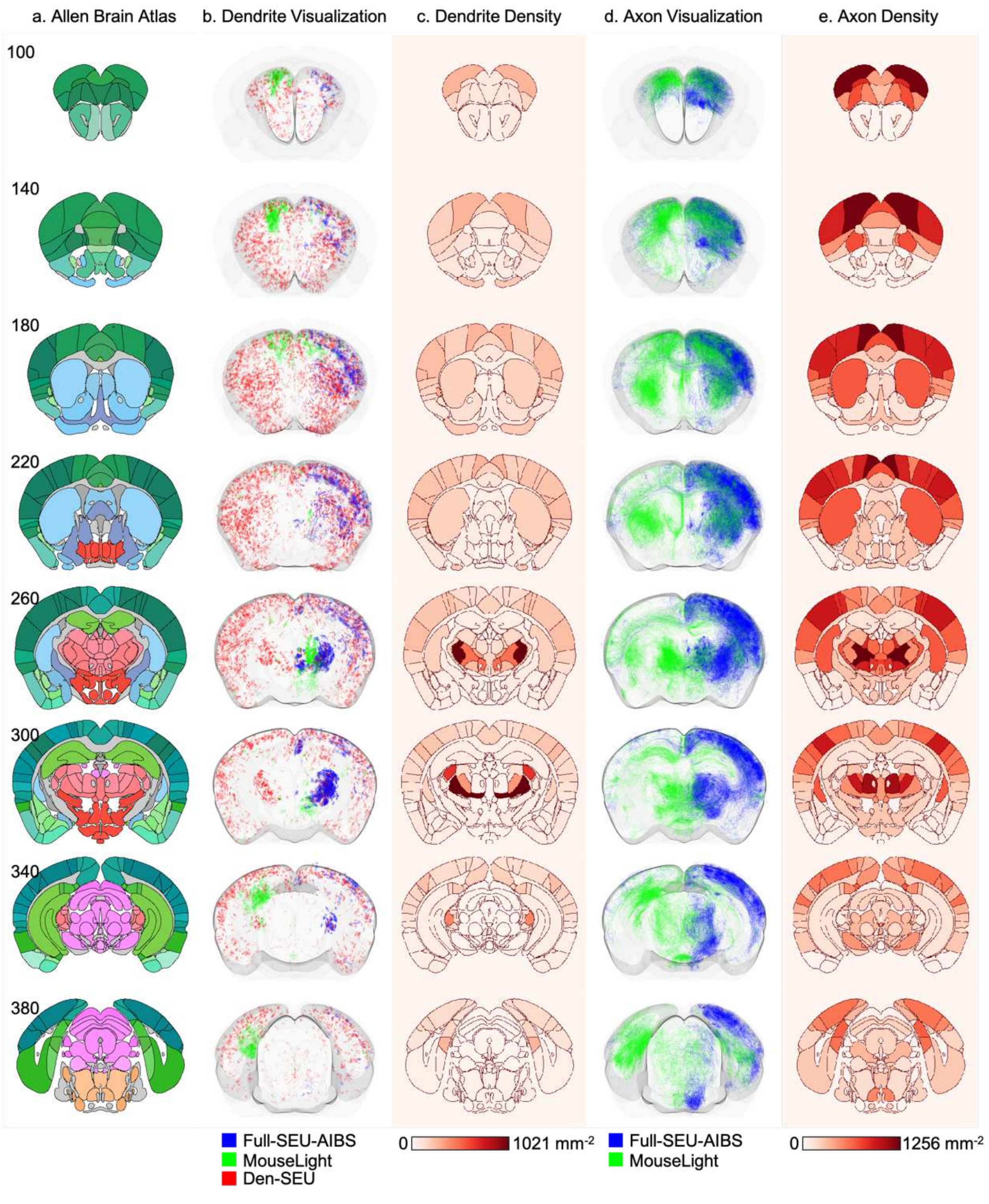

Supplementary Figure 1. Exemplar summary of the spatial anatomical distribution of several neuron reconstruction datasets. Eight axial slices are selected for visualization. (a) CCF atlas showing brain regions of selected slices. Brain regions are colored following CCF's color code. CCF slice ID is shown on the top-left of each image. (b) Visualization of dendrite reconstructions. (c) Dendrite density within each brain region. (d) Visualization of axonal arbor

reconstructions. (e) Axonal arbor density within each brain region. (b) and (d), In each image, dendrite/axonal arbors within 500 μm (20 slices) of the target slice are shown.
Different color is assigned to different dataset. (c) and (e), Density is computed by dividing total dendrite/axonal arbor length (mm) inside a brain region by the volumetric size (mm^3) of the brain region. The unit of arbor density is mm^{-2}. The color map is shown on the bottom.

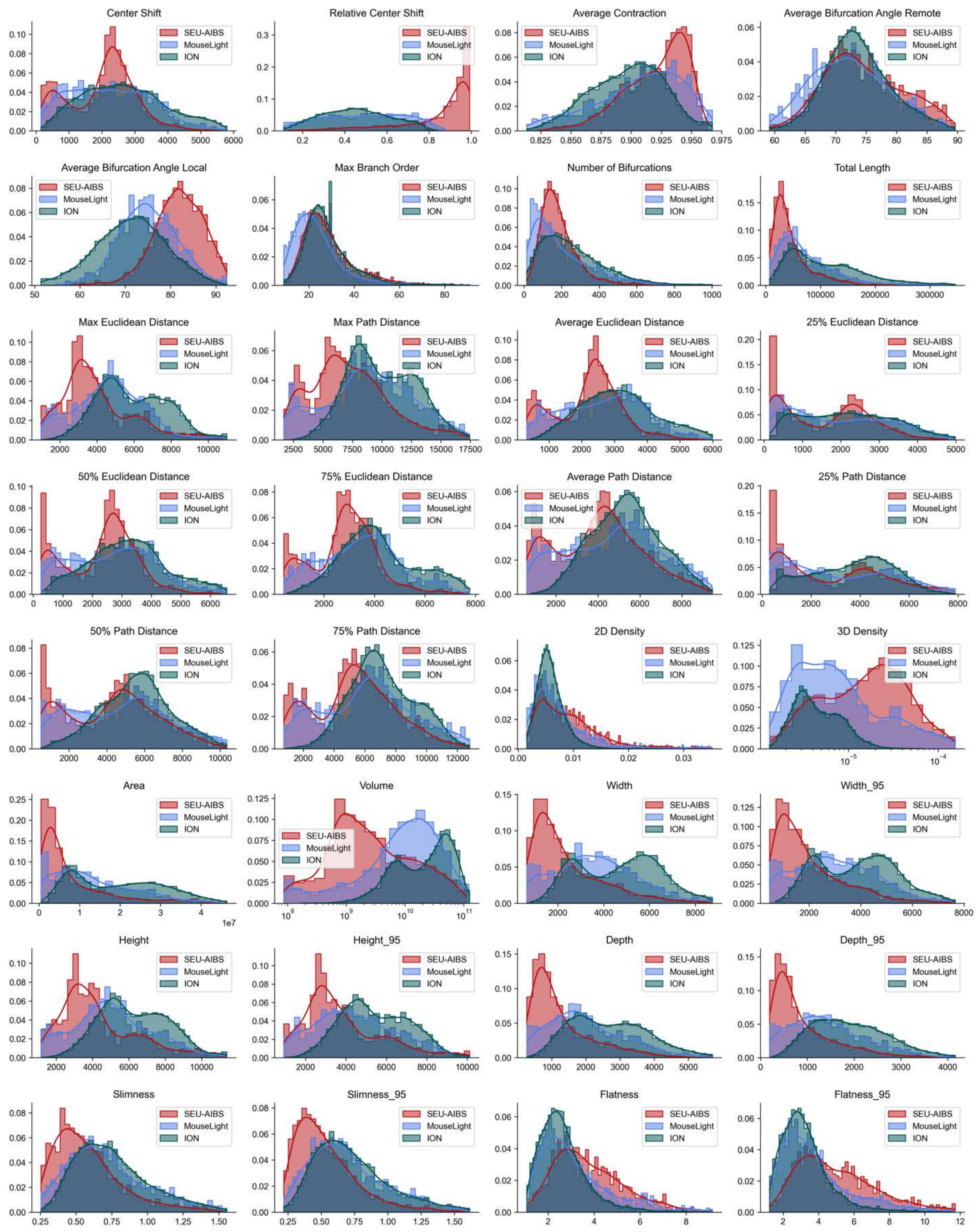

Supplemental Figure 2. Comparative analysis of morphological features of axons in three datasets, i.e. BICCN AIBS/SEU-ALLEN (SEU-AIBS), Janleia MouseLight (MouseLight), and ION.

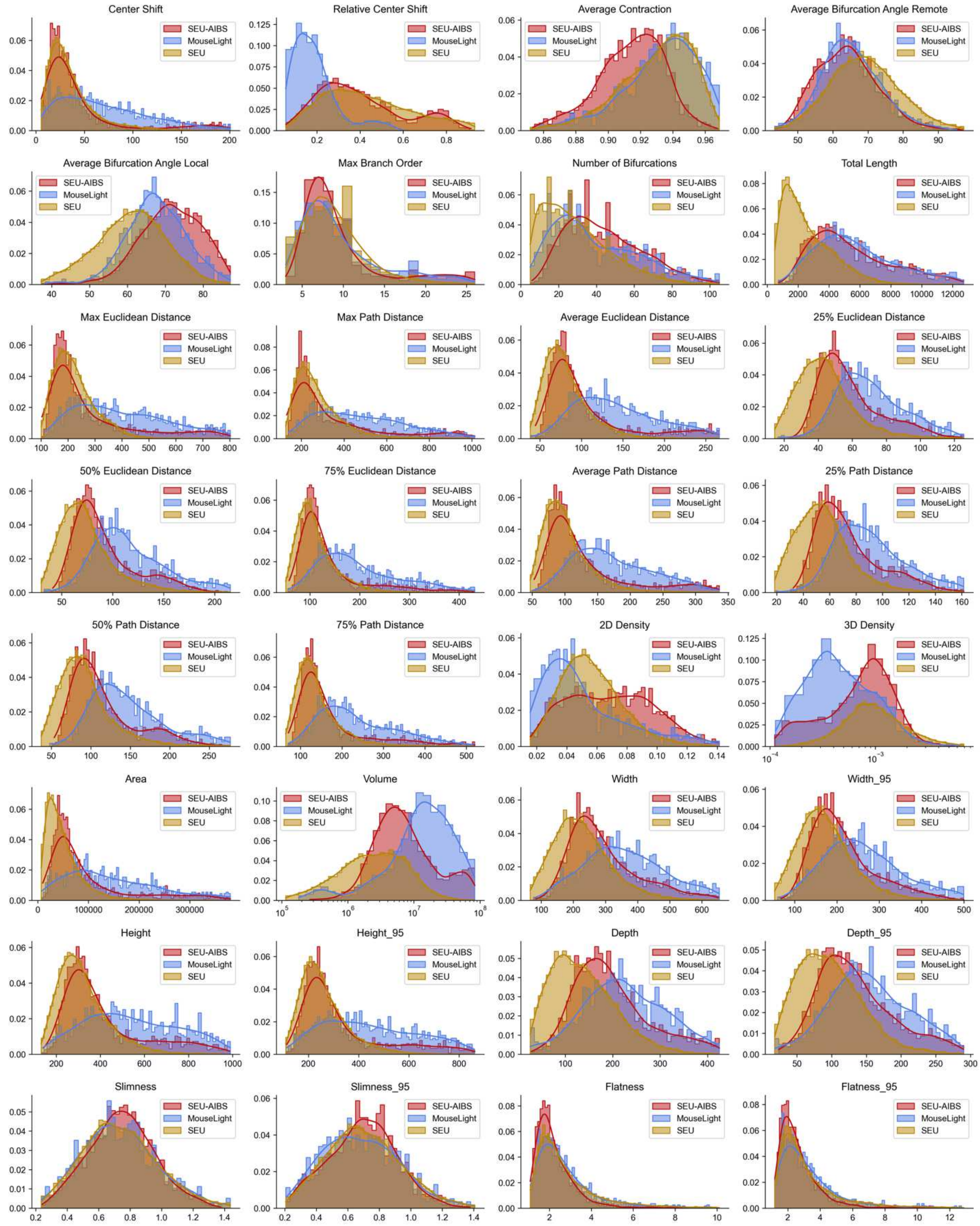

Supplemental Figure 3. Comparative analysis of morphological features of dendrites in three datasets, i.e. BICCN AIBS/SEU-ALLEN (SEU-AIBS), Janleia MouseLight (MouseLight), and DEN-SEU (SEU).

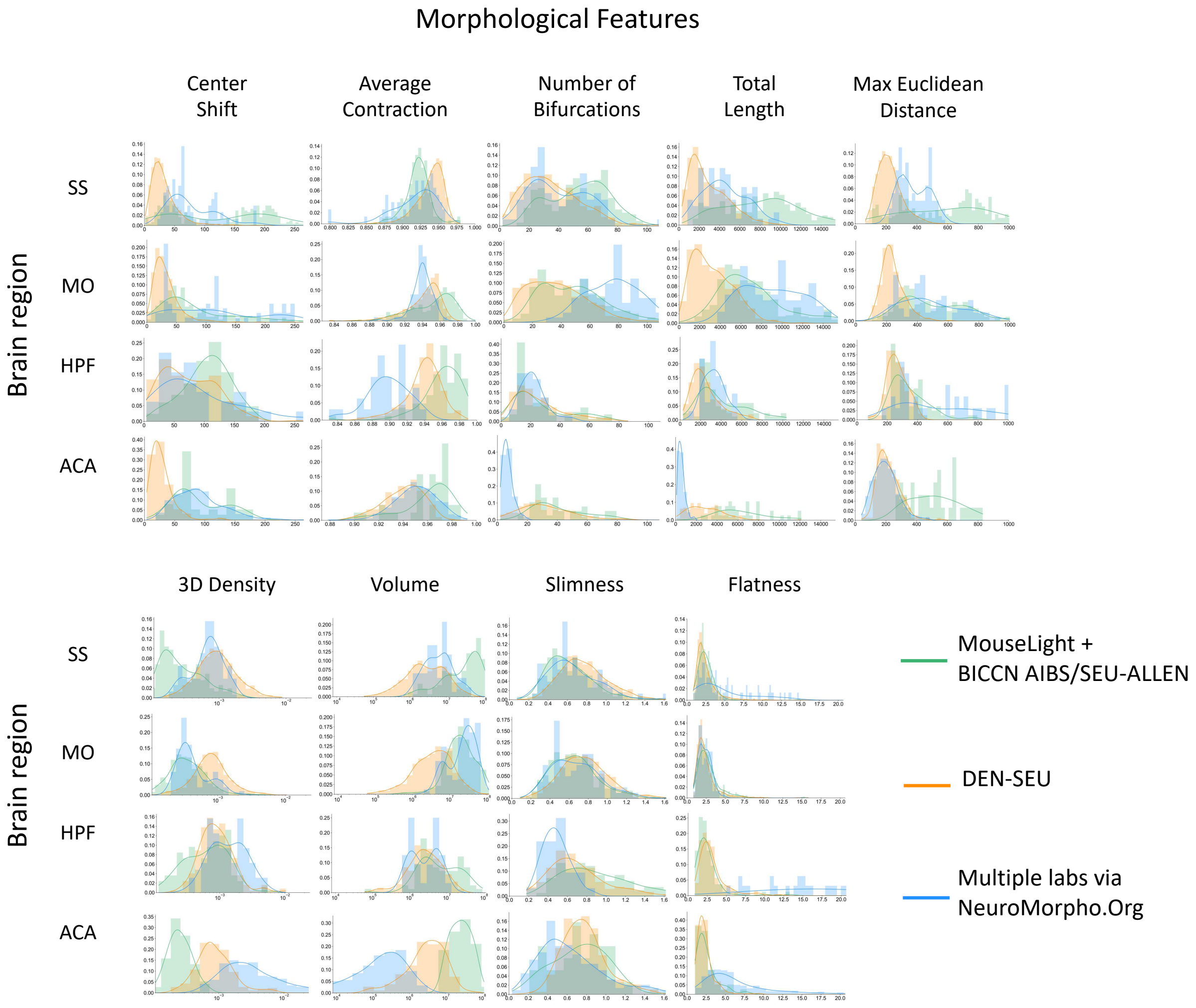

Supplemental Figure 4. Comparative analysis of dendrite morphology features of neurons in selected brain regions, for multiple datasets including full single neuron reconstructions (BICCN AIBS/SEU-ALLEN and MouseLight), dendritic reconstructions (DEN-SEU), and publicly available reconstructions from multiple independent labs (as archived at NeuroMorpho.Org, see **Methods**). Each row corresponds to a brain region, while each column corresponds to a feature. Four brain regions, i.e. somatosensory area (SS), somatomotor area (MO), hippocampal area (HPF), and anterior cingulate area (ACA), with available neuron feature data were selected. Nine informative features are shown as examples. Refer to **Supplementary Figure 5** for a complete comparison of all 32 features.

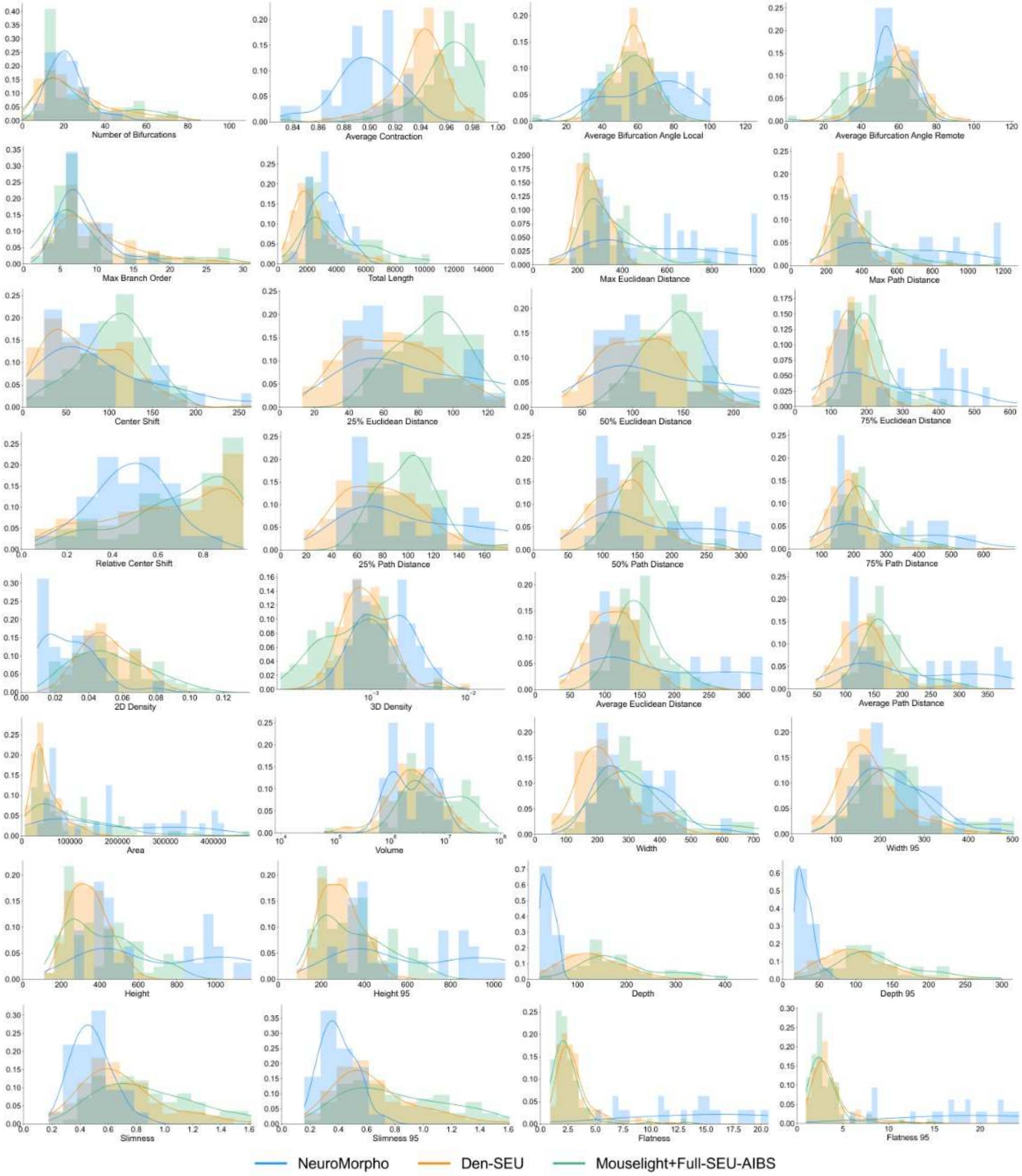

(a) somatosensory area (SS)

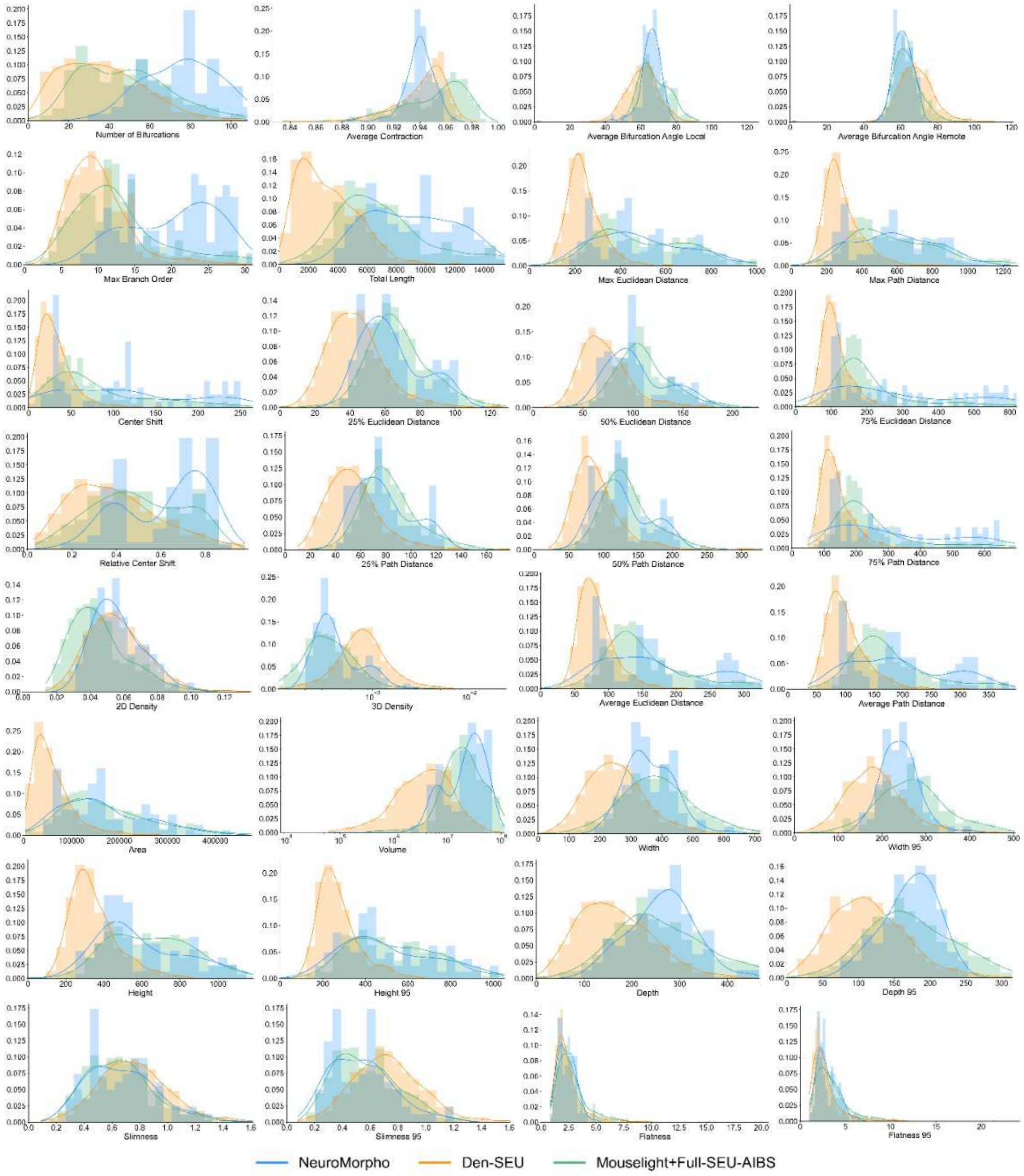

(b) somatomotor area (MO)

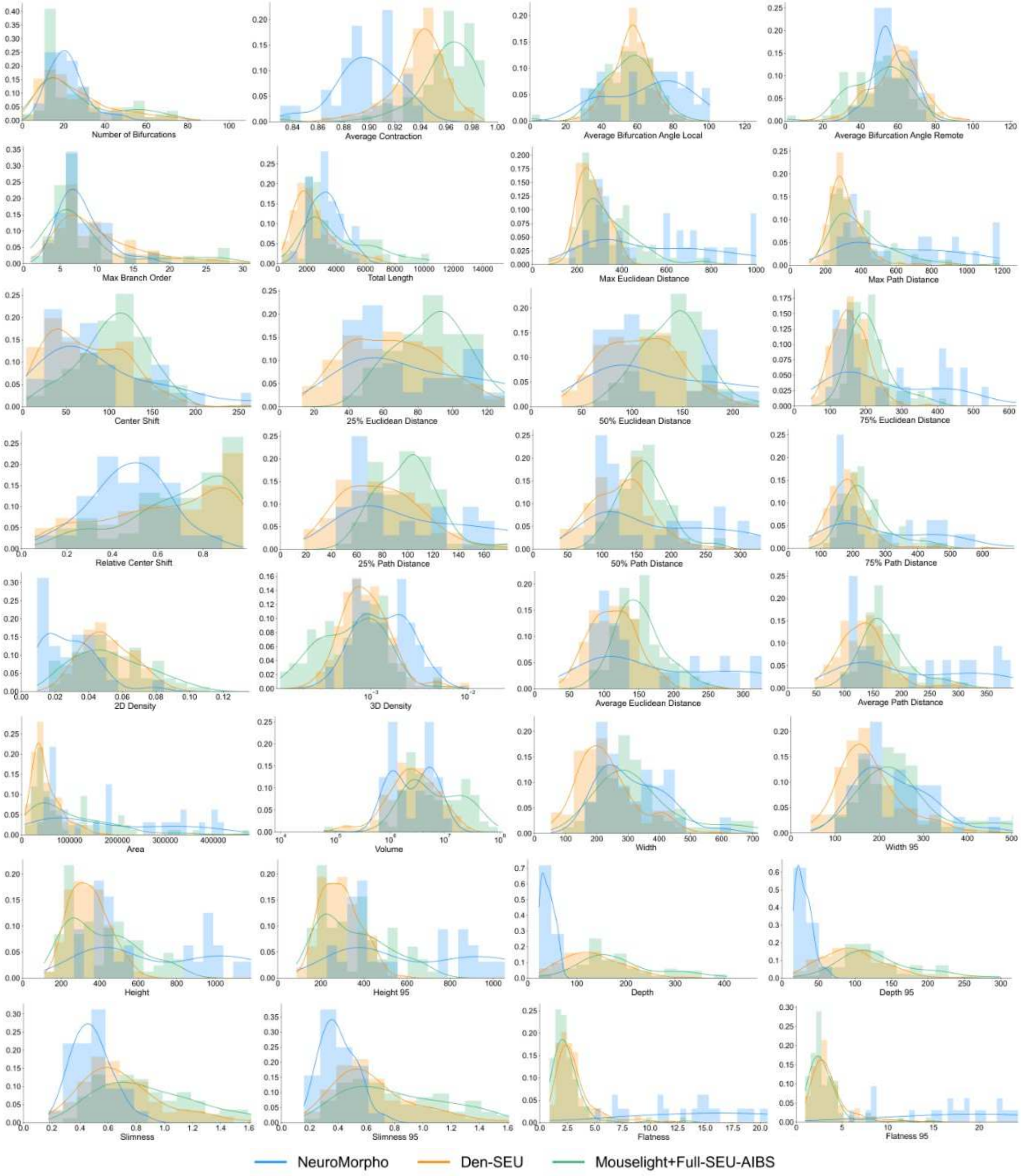

(c) hippocampal area (HPF)

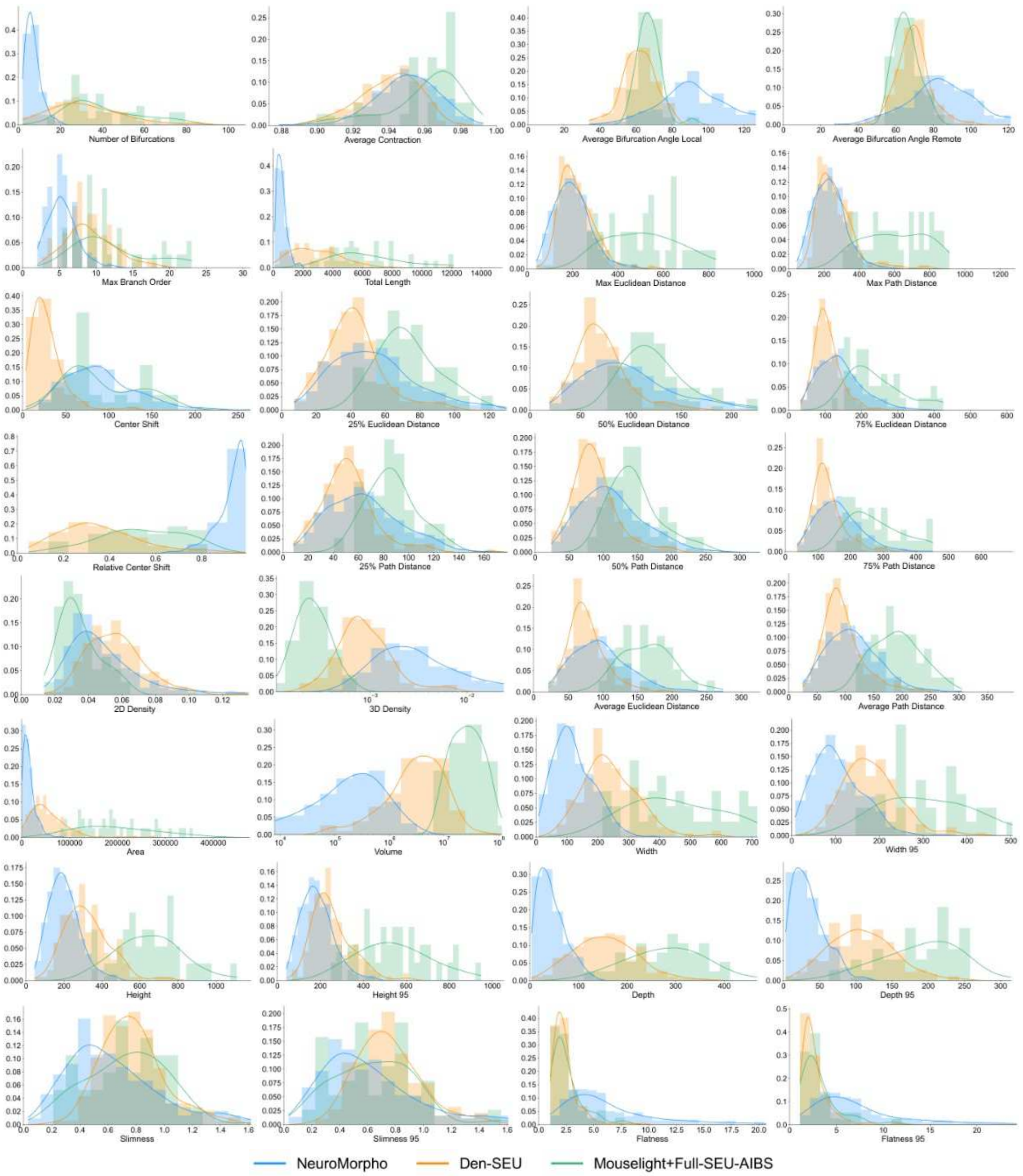

(d) anterior cingulate area (ACA)

Supplemental Figure 5. Comparative analysis of dendrite morphology features of neurons in selected brain regions, for multiple datasets including full single neuron reconstructions (BICCN AIBS/SEU-ALLEN and MouseLight, labeled as “MouseLight+Full-SEU-AIBS”), dendritic reconstructions (DEN-SEU/“Den-SEU”), and publicly available reconstructions from multiple independent labs (“NeuroMorpho”, as archived at NeuroMorpho.Org, see **Methods**). Four brain regions, i.e. (a) somatosensory area (SS), (b) somatomotor area (MO), (c) hippocampal area (HPF), and (d) anterior cingulate area (ACA), are shown with the comparison, in which all 32 morphology features are visualized with the respective names under each subplot.

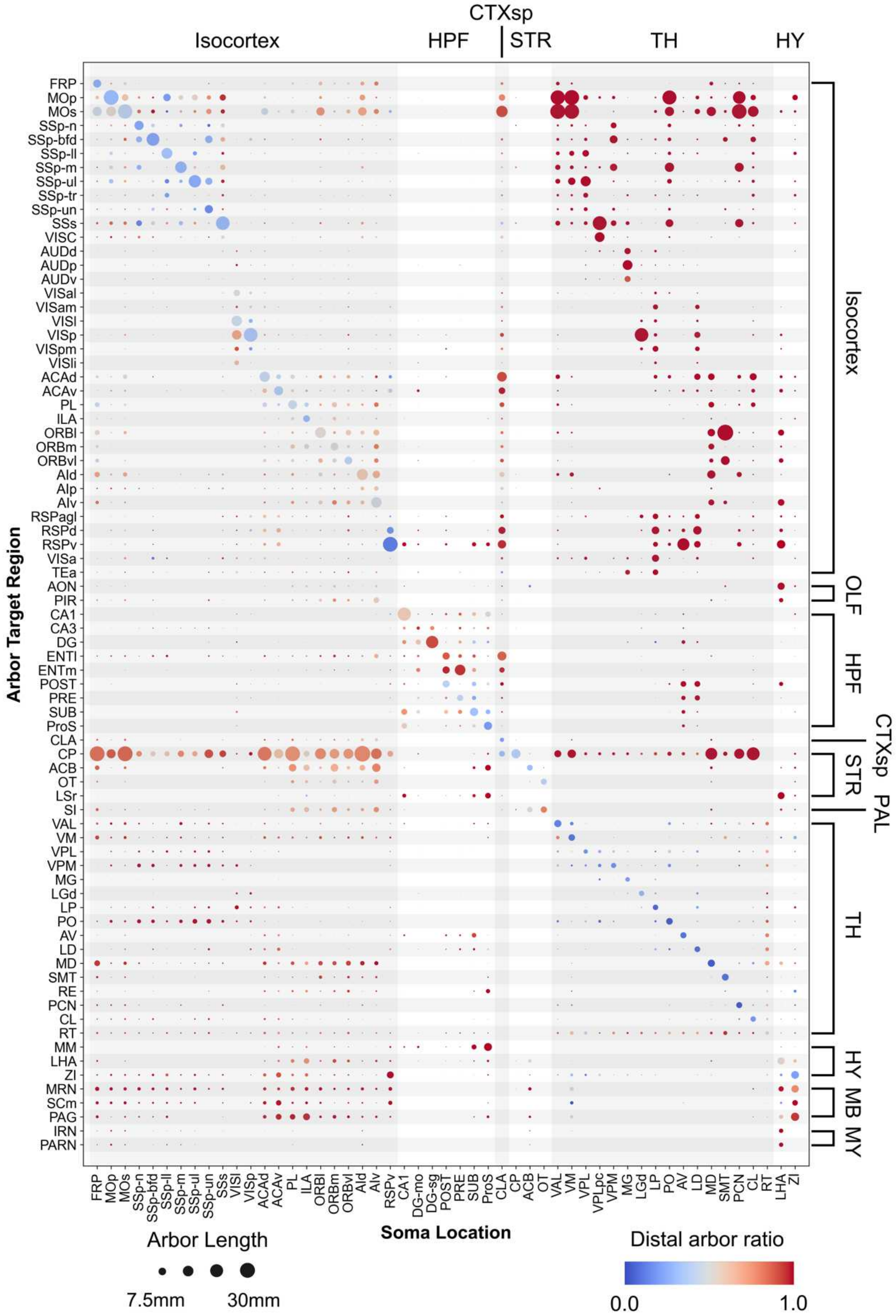

Supplemental Figure 6. Whole brain arborization map of all neurons with axons in this study. See **Figure 1D** for labels of brain regions.

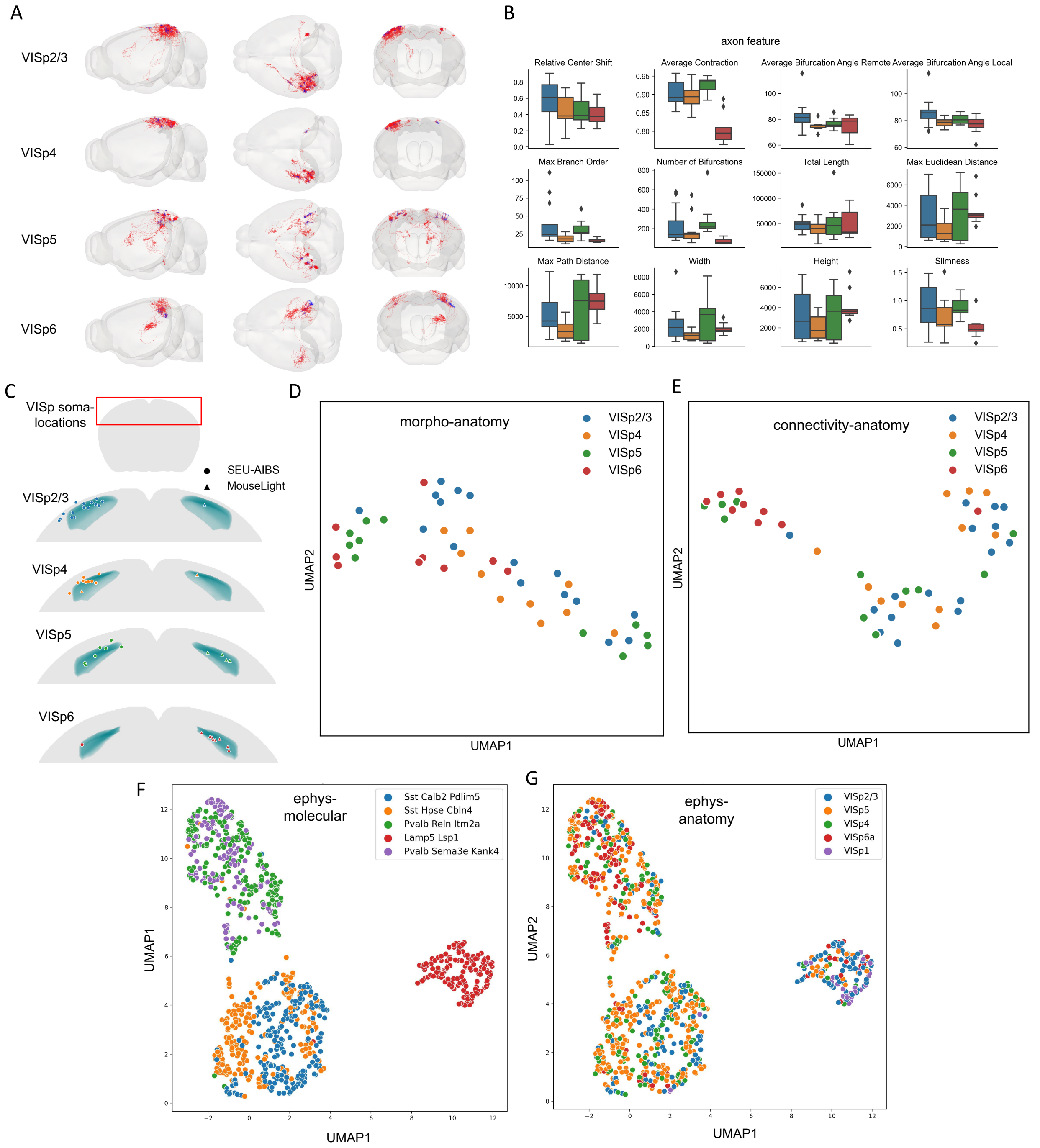

Supplemental Figure 7. Comparative analysis of projection patterns of VISp neurons originated in various cortical layers (2/3, 4, 5, and 6), the respective morphological features and soma locations, and public-domain electrophysiological recording and transcriptomic profiles of single neurons. **A**. Projection and regional connectivity patterns of VISp neurons, grouped by soma-locations in four cortical layers. **B**. Comparison of axon features of VISp neurons in four layers. **C**. Locations of VISp neurons used in this study. **D**. Joint distribution of morphological features and soma locations in the respective UMAP space. **E**. Joint distribution of connectivity features

and soma locations in the respective UMAP space. **F**. Joint distribution of electrophysiological features and molecular types of VISp neurons in the respective UMAP space. **G**. Joint distribution of electrophysiological features and soma locations in the respective UMAP space.

Supplementary Files

This is a list of supplementary files associated with this preprint. Click to download.

- STable1neurondatasummaryyzx20230414.docx
- STable2abbr02022023.csv
- STable3DataNeuroMorpho.docx
- STable4domainnames.csv
- STable5SupplementaryAxonMorphologicalFeatures.csv
- STable6SupplementaryDendriteMorphologicalFeatures.csv
- STable7old6etypefeatures.xlsx
- STable8old7projectioncluster.neuronclusterid.csv
- SFig1summaryofdatapic.png
- SFig2axonmorphofeature.png
- SFig3denmorphofeature.png
- sfig4comparedendritefeatures.pdf
- SFig50413ljl.docx
- SFig69298arborprojection.png
- SFig7etype.pdf
